# Microfluidic-Based Measurement Method of Red Blood Cell Aggregation under Hematocrit Variations

**DOI:** 10.3390/s17092037

**Published:** 2017-09-06

**Authors:** Yang Jun Kang

**Affiliations:** Department of Mechanical Engineering, Chosun University, 309 Pilmun-daero, Dong-gu, Gwangju 61452, Korea; yjkang2011@chosun.ac.kr; Tel.: +82-62-230-7052; Fax: +82-62-230-7055

**Keywords:** microfluidic device, disposable suction pump, periodic on-off blood delivery, ESR aggregation index (EAI), RBC aggregation index (AI), erythrocyte sedimentation rate (ESR)

## Abstract

Red blood cell (RBC) aggregation and erythrocyte sedimentation rate (ESR) are considered to be promising biomarkers for effectively monitoring blood rheology at extremely low shear rates. In this study, a microfluidic-based measurement technique is suggested to evaluate RBC aggregation under hematocrit variations due to the continuous ESR. After the pipette tip is tightly fitted into an inlet port, a disposable suction pump is connected to the outlet port through a polyethylene tube. After dropping blood (approximately 0.2 mL) into the pipette tip, the blood flow can be started and stopped by periodically operating a pinch valve. To evaluate variations in RBC aggregation due to the continuous ESR, an EAI (Erythrocyte-sedimentation-rate Aggregation Index) is newly suggested, which uses temporal variations of image intensity. To demonstrate the proposed method, the dynamic characterization of the disposable suction pump is first quantitatively measured by varying the hematocrit levels and cavity volume of the suction pump. Next, variations in RBC aggregation and ESR are quantified by varying the hematocrit levels. The conventional aggregation index (AI) is maintained constant, unrelated to the hematocrit values. However, the EAI significantly decreased with respect to the hematocrit values. Thus, the EAI is more effective than the AI for monitoring variations in RBC aggregation due to the ESR. Lastly, the proposed method is employed to detect aggregated blood and thermally-induced blood. The EAI gradually increased as the concentration of a dextran solution increased. In addition, the EAI significantly decreased for thermally-induced blood. From this experimental demonstration, the proposed method is able to effectively measure variations in RBC aggregation due to continuous hematocrit variations, especially by quantifying the EAI.

## 1. Introduction

Cardiovascular diseases including atherosclerosis [1,2], coronary heart disease [3], myocardial infarction, and stroke occur suddenly and without signs or symptoms, and eventually lead to serious complications or even death. Blood clotting or abnormal blood flow may cause vascular blockage, which prevents blood from flowing to the heart or brain. For this reason, biochemical analyses, such as biomarkers in cardiovascular diseases and disorders [4] or DNA [5], do not show sufficient promise for the early detection of cardiovascular diseases [6]. Since the associations between coronary heart diseases and blood rheology have been sufficiently investigated [3,7,8], several biophysical properties such as the viscosity [9,10,11,12,13,14,15,16], erythrocyte sedimentation rate (ESR) [17,18,19,20,21,22], hematocrit [23,24,25], aggregation [26,27,28,29,30,31,32], and deformability [33,34,35,36,37,38,39,40] are employed to detect variations in blood samples. Among them, blood viscosity varies depending on several factors, such as the plasma viscosity, hematocrit, red blood cell (RBC) aggregation, and deformability. At high shear rates, blood viscosity decreases, owing to the deformation and alignment of RBCs in blood flows. At lower shear rates, RBC aggregation contributes to increasing blood viscosity.

Recently, a microfluidic platform has been widely employed to measure blood viscosity due to several advantages such as short measurement time, small volume consumption, and disposability. Using a syringe pump, blood viscosity is quantified by monitoring blood flows at a consistent flow rate. Since the syringe pump might supply a consistent flow rate at higher flow rates, most microfluidic-based viscometers are employed to measure blood viscosity at a relative higher shear rate ($\dot{\gamma }$) (i.e., $\dot{\gamma }$ > 10 s^−1^) [14,41]. However, at lower shear rates, a microfluidic-based viscometer does not provide consistent blood viscosity because of fluidic instability of syringe pump [42]. At lower shear rates (i.e., $\dot{\gamma }$ = 1–10 s^−1^) [43], RBC aggregates in a stack-of-coins form, which varies depending on several factors such as plasma proteins, membrane flexibility, and the hematocrit [44]. The presence of fibrinogen or polymer dextran leads to RBC aggregation, and stabilizes RBC clusters in microcapillary flows [31,45]. A linear viscoelasticity property is also suggested to evaluate the effect of hematocrit or dextran on RBC aggregation [46].

Thus, instead of blood viscosity, RBC aggregation in a microfluidic platform is considered significant for monitoring the variations in blood rheology, especially at extremely low shear rates. 

When dropping a small volume of blood into the inlet port of a microfluidic device, the blood tends to fill all microfluidic channels. After a certain amount of time, blood flow stops and aggregates in rouleaux form. Then, RBC aggregation is measured by quantifying the temporal variations of image intensity [17] or electric impedance [18] (i.e., a syllectogram [20]). A repetitive measurement is conducted after agitating blood flow with a pinch valve [17]. Here, the hematocrit of blood is kept constant during the experiment. However, when delivering blood into a microfluidic device with external sources, including a pressure source [26,30] or syringe pump [19,28,47], ESR (erythrocyte sedimentation rate) occurs continuously in a tube [19] or reservoir [26]. In other words, the RBCs migrate toward the bottom, in the gravitational direction, because of the gravity force. The RBC-depleted region (or plasma region) expands from the top to the bottom region in a tube [19]. Thus, the hematocrit tends to increase at the bottom region of a tube. For this reason, if a blood sample is supplied to a microfluidic device from the bottom region of the tube, the hematocrit of blood varies continuously due to the ESR in the tube [19]. However, most of the previous methods measure RBC aggregation with a conventional aggregation index (AI), which does not account for the hematocrit variations owing to the ESR. Thus, a new method should be devised to consider the effect of hematocrit variation on RBC aggregation in a microfluidic device. In addition, a bulk-sized syringe pump should be replaced by a disposable suction pump for promising point-of-care testing (POCT). 

In this study, a microfluidic-based measurement technique is suggested to measure RBC aggregation under the varying hematocrit caused by the continuous ESR in a tube. To demonstrate the proposed method, the dynamic characterization of the disposable suction pump is first quantitatively measured by varying two factors, including the hematocrit (H_ct_) (H_ct_ = 10%, 20%, 30%, 40%, and 50%), and cavity volume (V_cavity_) (V_cavity_ = 0.1 mL, 0.2 mL, and 0.3 mL). Second, the RBC aggregation and ESR are quantified by varying the hematocrit. Finally, the proposed method is applied to detect the effect of a specific dextran solution and heat-treated blood on the RBC aggregation and ESR, respectively.

## 2. Experimental Section

### 2.1. Microfluidic-Based Measurement of RBC Aggregation under Continuous ESR in a Pipette Tip

A microfluidic-based measurement method is suggested to measure variations of RBC aggregation due to the continuous ESR in a pipette tip. Since RBCs aggregate and stack in a pipette tip, the hematocrit of blood supplied into a microfluidic channel varies with an elapse of time. Thus, variations of RBC aggregation owing to the ESR in a pipette tip are quantified by monitoring the temporal variations of image intensity within a specific region of interest (ROI) in a microfluidic channel. 

**Figure 1:**
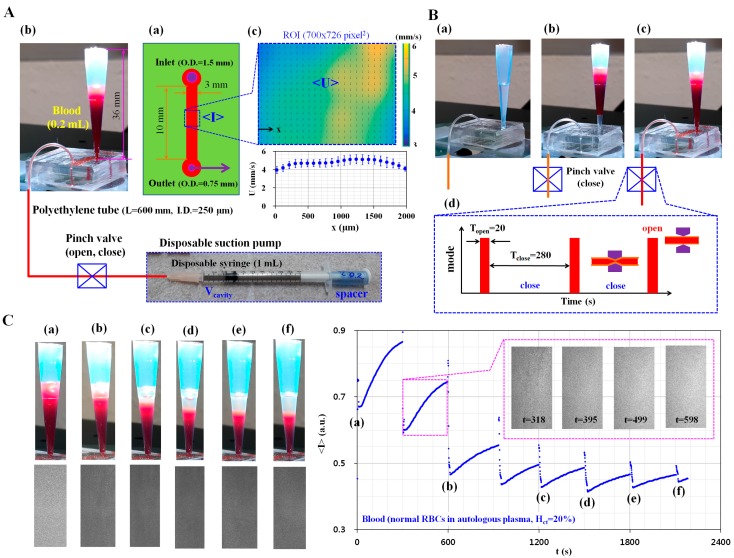
Schematics for measuring variations of red blood cell (RBC) aggregation owing to the erythrocyte sedimentation rate (ESR) in a pipette tip under periodic on–off blood delivery with a disposable suction pump. (**A**) Schematic of the experimental setup, including a disposable suction pump and a disposable microfluidic device with a pipette tip; (**a**) The microfluidic device; (**b**) The pipette tip tightly fitted into the inlet port, a disposable suction pump, and a pinch valve; (**c**) Averaged blood velocity (<U>) and averaged image intensity (<I>) over the specific region of interest (ROI); (**B**) Blood delivery procedure from the pipette tip to the microfluidic channel; (**a**) The microfluidic device and the tube are filled with a 1% BSA solution; (**b**) After removing the 1% BSA solution, the blood is dropped into the pipette tip; (**c**) Using the disposable suction pump, the microfluidic channel and the tube are filled with blood; (**d**) Operation of the pinch valve (open, close); (**C**) As a preliminary demonstration, to evaluate the variation of the RBC-depleted region owing to ESR in the pipette tip, snapshots are captured at a specific duration of time (t) [(**a**) t = 0; (**b**) t = 600 s; (**c**) t = 1200 s; (**d**) t = 1500 s, (**e**) t = 1800 s; and (**f**) t = 2100 s]. Inset shows microscopic images captured at a specific time (t) (t = 318 s, 395 s, 499 s, and 598 s).

Figure 1 shows the schematics for measurement of RBC aggregation owing to ESR in a pipette tip, under blood delivery with a disposable suction pump. As depicted in Figure 1A, the experimental setup is composed of a disposable microfluidic device with a pipette tip, a disposable suction pump, and a pinch valve (Supa clip, Pankyo, Korea). The microfluidic device is designed with an inlet port, a single microfluidic channel (width = 3 mm, length = 10 mm, and depth = 100 µm), and an outlet port (Figure 1A(a)). To connect with the pipette tip (~1 mL) and polyethylene tube, the inlet and outlet ports have 1.5 mm and 0.75 mm holes, respectively. After cutting approximately 36 mm of a pipette tip, it is tightly fitted into the hole of the inlet port (Figure 1A(b)). After fitting into the hole of the outlet port, the polyethylene tube (inner diameter = 250 µm, length = 600 mm) is connected to the disposable suction pump. The disposable suction pump is composed of a disposable syringe (~1 mL), a specifically sized spacer, and a pinch valve. To secure the cavity volume (V_cavity_) of the disposable suction pump, the polyethylene tube is clamped with a pinch valve. After the plunger is sucked outward sufficiently, the specific spacer for the corresponding cavity volume is inserted between the stopper and plunger. After a blood sample (~0.2 mL) is dropped into the pipette tip with a pipette, the blood flow stops or runs in the microfluidic channel by clamping or releasing the tube with the pinch valve. 

To monitor the variations of blood velocity and image intensity, two microscopic images are captured consecutively at a frame rate of 1 kHz by using a high-speed camera (FASTCAM MINI, Photron, San Diego, CA, USA) triggered by a function generator at an interval of 1 s. After selecting a specific ROI (700 × 729 pixels) along the microfluidic channel, the velocity field (U) and image intensity (I) are obtained by conducting time-resolved micro-PIV (Particle Image Velocimetry) and digital image processing, respectively. Thereafter, the averaged blood velocity (<U>) and averaged image intensity (<I>) are calculated by conducting an arithmetic average over the specific ROI (Figure 1A(c)). Figure 1B shows the blood delivery procedure from the pipette tip to the microfluidic channel. First, the microfluidic device and tube are filled with a 1% bovine serum albumin (BSA) solution to suppress non-specific binding with the plasma proteins (Figure 1B(a)). After removing the 1% BSA solution, blood (~0.2 mL) is dropped into the pipette tip with the pipette (Figure 1B(b)). By releasing the pinch valve, the microfluidic channel and tube are filled with blood (Figure 1B(c)). After then, by periodically closing or opening the pinch valve, the blood flow runs (T_open_ = 20 s) and stops for 280 s (T_close_ = 280 s) for a single period of 300 s (Figure 1B(d)). As a preliminary demonstration, the hematocrit of blood was adjusted to 20% by adding normal RBCs into autologous plasma. As shown in Figure 1C, to evaluate the variations in the RBC-depleted region (or plasma region) owing to the ESR in the pipette tip, snapshots were captured at specific times (t) [(a) t = 0, (b) t = 600 s, (c) t = 1200 s, (d) t = 1500 s, (e) t = 1800 s, and (f) t = 2100 s]. In addition, as shown in Figure S3, sequential snapshot images were captured with a smartphone-based camera at intervals of 30 s after clamping the tube with the pinch valve. The inset shows microscopic images captured at a specific time (t) (t = 318 s, 395 s, 499 s, and 598 s). The top surface of the blood filled in the pipette tip was gradually lowered due to the periodic releasing of the pinch valve. The continuous ESR in the pipette tip leads to the expansion of the RBC-depleted region (or plasma region) over time. In addition, temporal variations of image intensity were obtained. The minimum value of averaged image intensity (<I>_min_) for each period decreased abruptly down up to 900 s. Thereafter, <I>_min_ remains constant over time. In addition, the variation in amplitude of image intensity (VA) (i.e., VA = <I>_max_−<I>_min_) for each period decreased gradually with time. From this preliminary demonstration, it is estimated that the suggested method is able to detect variations of RBC aggregation owing to the ESR in a pipette tip.

### 2.2. Fabrication of the Microfluidic Device and Experimental Procedure

The microfluidic device was designed with an inlet port, an outlet port, and a single microfluidic channel (width = 3 mm, length = 10 mm, and depth = 100 µm). A silicon-master mold was fabricated with conventional microelectromechanical-system (MEMS) fabrication techniques such as photolithography and deep reactive ion etching (DRIE). To conduct soft lithography, polydimethylsiloxane (PDMS) (Sylgard 184, Dow Corning, Midland, MI, USA) was mixed at a 1:10 ratio of curing agent to PDMS monomer. Then, the PDMS mixture was poured into a silicon-master mold located in a petri dish. Air bubbles dissolved in the PDMS were completely removed by operating a vacuum pump for 1 h. After curing the PDMS in a convection oven at 80 °C for 2 h, a PDMS block was peeled off from the silicon-master mold. The PDMS block was cut with a razor blade. The inlet and outlet ports of the PDMS block were punched using two biopsy punches (outer diameter = 1.5 mm and 0.5 mm). After treating the PDMS block and a glass substrate with oxygen plasma (CUTE-MPR, Femto Science Co., Yongin, Korea), the microfluidic channel was closed by bonding the PDMS block to a glass slide. A pipette tip was cut approximately 36 mm from the bottom surface (Figure 1A(b)). After fitting the pipette tip into the inlet port of the microfluidic device, one end of the polyethylene tube (inner diameter = 250 µm, length = 600 mm) was inserted into the outlet port. The other end of the polyethylene tube was connected to the disposable suction pump. The disposable suction pump was prepared by assembling a disposable syringe (1 mL, BD Bioscience, Singapore), pinch valve, and specific-sized spacer for the corresponding cavity volume (Figure S1A). 

The microfluidic device was then placed on an optical microscope (IX53, Olympus, Tokyo, Japan) equipped with a 4× objective lens (NA = 0.1). After the 1% BSA solution was dropped into the pipette tip, the microfluidic channel and tube were filled with the 1% BSA solution by using the disposable suction pump. After 10 min, the BSA solution was removed with the disposable syringe. To measure RBC aggregation and ESR, blood (~0.2 mL) was dropped into the pipette tip. By opening or closing the pinch value, blood was delivered for 20 s and stopped for 280 s, for each period of 300 s. All experiments were conducted at a consistent room temperature of 25 °C.

### 2.3. Sample Preparation of Blood

Following the guidelines of the ethics committee in Chosun University Hospital (CUH), all experiments were performed while ensuring that the procedures were appropriate and humane. To simulate RBC aggregation or ESR, various blood samples were prepared by adding human RBCs to autologous plasma, or a specific concentration of dextran solution. The human RBCs were provided by the Gwangju–Chonnam Blood Bank (Gwangju, Korea). First, to evaluate the effect of the hematocrit on RBC aggregation and ESR, the hematocrit of normal RBCs (H_ct_) (H_ct_ = 10%, 20%, 30%, 40%, and 50%) was prepared by adding normal RBCs into autologous plasma. Second, to elevate the RBC aggregation and ESR in the pipette tip, four different concentrations of dextran solution (C_Dextran_) (C_Dextran_ = 5 mg/mL, 10 mg/mL, 15 mg/mL, and 20 mg/mL) were prepared by diluting dextran (*Leuconostoc* spp., MW = 450–650 kDa, Sigma-Aldrich, St. Louis, MO, USA) with a 1× PBS solution (pH 7.4, GIBCO, Life Technologies, Seoul, Korea). The hematocrit of blood was adjusted to 30% by adding normal RBCs to a specific dextran solution. Finally, a blood sample was prepared by adding normal RBCs into autologous plasma. After dropping them into a plastic bottle (~5 mL), the plastic bottle was moved into a convection oven. The temperature was set to 50 °C. After a certain amount of time (t) (t = 10 min, 20 min, and 30 min), the plastic bottle was then cooled down to the temperature of 25 °C. 

### 2.4. Quantification of Blood Velocity and Image Intensity

First, to evaluate the temporal variations of blood flow, velocity fields were obtained by conducting a time-resolved micro-PIV technique. As represented in Figure 1A(c), a specific ROI (700 × 729 pixels) along the microfluidic channel was selected to obtain velocity fields of blood flow. The size of the interrogation window was 32 × 32 pixels. Window overlap was 50%. The obtained velocity fields were validated with a median filter. The average velocities of blood flow (<U>) was calculated as an arithmetic average over the ROI. 

Second, to evaluate variations in the image intensity of blood flow, a specific ROI (700 × 729 pixels) was selected along the microfluidic channel, as shown in Figure 1A(c). The averaged image intensity over the ROI (<I>) was calculated by performing digital image processing with commercial software (Matlab, Mathworks, Natick, MA, USA). 

## 3. Results and Discussion

### 3.1. Dynamic Characterization of a Disposable Suction Pump

**Figure 2:**
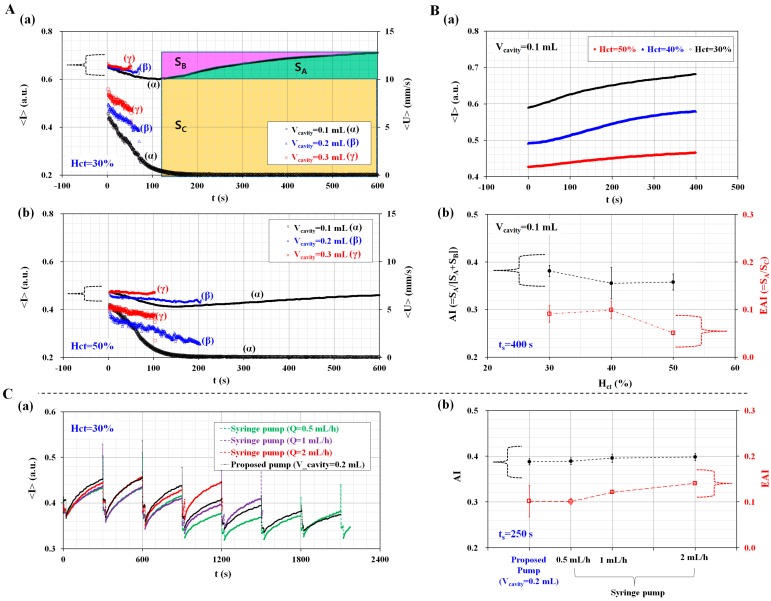
Dynamic characterization of the disposable suction pump with respect to hematocrit (H_ct_) and cavity volume (V_cavity_). (**A**) Temporal variations of the averaged image intensity (<I>) and averaged blood velocity (<U>) with respect to H_ct_ and V_cavity_; (**a**) Temporal variations of <I> and <U> for blood (30% hematocrit) by varying the cavity volume. Three factors (S_A_, S_B_, and S_C_) are calculated from the temporal variations of <I>; (**b**) Temporal variations of <I> and <U> for blood (50% hematocrit) with respect to V_cavity_; (**B**) Quantitative evaluation of the aggregation index (AI) and Erythrocyte-sedimentation-rate Aggregation Index (EAI) with respect to H_ct_; (**a**) Temporal variations of <I> were obtained by varying the hematocrit (H_ct_) (H_ct_ = 30%, 40%, and 50%) at a fixed cavity volume of V_cavity_ = 0.1 mL. Temporal variations of <I> are obtained after resetting t = 0 at a minimum value of <I> (Figure 2A(a)); (**b**) Variations of the AI and EAI with respect to hematocrit. Time duration (t_s_) is fixed at t_s_ = 400 s for calculating the three factors. Using the three factors, the AI (i.e., RBC aggregation effect) and EAI (i.e., ESR effect) are calculated as AI = S_A_/(S_A_ + S_B_) and EAI = S_A_/S_C_, respectively; (**C**) Performance comparison between proposed pump and commercial syringe pump; (**a**) Temporal variations of averaged image intensity (<I>) depending on the proposed pump and syringe pump; (**b**) Variations of AI and EAI with respect to proposed method (i.e., V_cavity_ = 0.2 mL) and syringe pump (i.e., Q = 0.5 mL/h, 1 mL/h, and 2 mL/h).

The dynamic characterization of a disposable suction pump was evaluated by varying the hematocrit (H_ct_) and cavity volume (V_cavity_). Previously, microfluidic-integrated pumps were operated using a bulk-sized facility [48,49]. In this study, to deliver blood in an easy and compact way, a disposable suction pump was prepared by assembling a disposable syringe, spacer, and pinch valve. In addition, to stop blood flow quickly, a pinch valve was installed in front of a disposable syringe. To secure the cavity volume, a spacer for the corresponding cavity volume was prepared by cutting L = 17.5 mm (V_cavity_ = 0.1 mL), L = 23.8 mm (V_cavity_ = 0.2 mL), and L = 29.2 mm (V_cavity_ = 0.3 mL) from the top surface (Figure S1A). After the tube was clamped with a pinch valve, the plunger was sucked outward sufficiently. Subsequently, the corresponding spacer for each cavity volume was inserted between the plunger and stopper. As shown in Figure S1B, three disposable suction pumps were prepared for the corresponding cavity volume (V_cavity_) (V_cavity_ = 0.1 mL, 0.2 mL, and 0.3 mL). To evaluate the variations of blood flow with respect to cavity volume (V_cavity_), the pinch valve was released during this test. Figure 2A shows the temporal variations of <I> and <U> with respect to the hematocrit and cavity volume. The hematocrit of blood was adjusted to H_ct_ = 30%, 40%, and 50% by adding normal RBCs into autologous plasma. At 30% hematocrit (H_ct_ = 30%), the temporal variations of <I> and <U> were obtained by varying the cavity volume (V_cavity_) (V_cavity_ = 0.1 mL, 0.2 mL, and 0.3 mL), as shown in Figure 2A(a). As a result, blood velocity (<U>) increased when the cavity volume enlarged, and <U> decreased gradually with time. The quantification was continued until blood filled the microfluidic channel. In other words, 0.2 mL blood was supplied for 53 s (V_cavity_ = 0.3 mL) and 71 s (V_cavity_ = 0.2 mL), respectively. However, for the disposable suction pump with V_cavity_ = 0.1 mL, the blood flow was maintained in the microfluidic channel for over 140 s. Depending on blood velocity in the microfluidic channel, image intensity (<I>) decreased over time. For V_cavity_ = 0.1 mL, <I> decreased gradually by up to 140 s. After 140 s, the blood flow stopped. It is estimated that increases in <I> were due to RBC aggregation. Using the temporal variations of <I> for V_cavity_ = 0.1 mL (i.e., a syllectogram), three factors (S_A_, S_B_, and S_C_) were calculated and represented in the inset of Figure 2A(a). Here, a syllectogram is denoted as the temporal variations of an intensity plot. The three factors were calculated using the following expressions,

(1) $$S_{A}=\int_{0}^{t_{s}}\left(<I>-<I{>}_{\min}\right)\;\mathrm{dt}$$

(2) $$S_{B}=\int_{0}^{t_{s}}\left(<I{>}_{\max}-<I>\right)\;\mathrm{dt}$$

(3) $$S_{C}=\int_{0}^{t_{s}}<I{>}_{\min}\;\mathrm{dt}$$

In Equations (1)–(3), t_s_ denotes integral time. In addition, <I>_min_ and <I>_max_ denote <I (t = 0)> and <I (t = t_s_)>, respectively. At 50% hematocrit (H_ct_ = 50%), the temporal variations of <I> and <U> were obtained by varying the cavity volume, as shown in Figure 2A(b). From this result, a higher value of the hematocrit (H_ct_ = 50%) leads to a significant decrease in <I> and <U>, compared with a lower value of the hematocrit (H_ct_ = 30%). In addition, for V_cavity_ = 0.1 mL, <I>_min_ and VA are significantly decreased, compared with a lower value of the hematocrit (H_ct_ = 30%). Blood with 30% hematocrit has much larger <I>_min_ and VA than blood with 50% hematocrit, due to RBC aggregations and ESR in the pipette tip.

The RBC aggregation and ESR were obtained by varying the hematocrit levels (H_ct_) (H_ct_ = 30%, 40%, and 50%) through using the syllectogram obtained by using the disposable suction pump with V_cavity_ = 0.1 mL. As shown in Figure 2B(a), temporal variations of (<I>) were obtained by increasing the hematocrit (H_ct_). Here, temporal variations of (<I>) were obtained after resetting t = 0 at a minimum value of (<I>). Three factors (S_A_, S_B_, and S_C_) were obtained by analyzing the temporal variations of (<I>). Then, the RBC aggregation and ESR were quantified by calculating two indices: AI, and a newly devised index called the Erythrocyte-sedimentation-rate Aggregation Index (EAI). Here, AI is considered as the conventional aggregation index, and calculated as AI = S_A_/(S_A_ + S_B_) [20]. On the other hand, EAI is a new index to consider variations of RBC aggregation due to the ESR in a pipette tip. In other words, the ESR in a pipette tip leads to an increased hematocrit of the blood that was delivered into the microfluidic channel. Because RBC aggregation is significantly influenced by the hematocrit, the RBC aggregation was varied with time. Thus, to evaluate the variations of RBC aggregation owing to ESR, the EAI is newly defined as the ratio of S_A_ (i.e., the RBC aggregation effect) to S_C_ (i.e., the ESR effect) (i.e., EAI = S_A_/S_C_). As shown in Figure S2, variations of AI and EAI were obtained by varying integral time (t_s_). Both AI and EAI are increased gradually by increasing t_s_. For convenience, time duration (t_s_) was fixed at t_s_ = 400 s for calculating the three factors. As shown in Figure 2B(b), variations of AI and EAI were obtained by varying the hematocrit (H_ct_) (H_ct_ = 30%, 40%, and 50%). As a result, because AI remains unrelated to the hematocrit, the hematocrit does not contribute to the varying RBC aggregation. However, blood with a higher hematocrit (H_ct_ = 50%) has a lower value of EAI, compared with blood with a lower hematocrit (H_ct_ = 30%). At last, the performance of the proposed pump was compared with that of a commercial syringe pump (neMESYS, Centoni Gmbh, Korbußen, Germany). Here, the hematocrit of the blood was adjusted to 30% by adding normal RBCs into autologous plasma. To evaluate the variations of AI and EAI with respect to the flow rate of the syringe pump, flow rate (Q) was set to Q = 0.5 mL/h, 1 mL/h, and 2 mL/h as withdrawal mode. 

Immediately, with the operation of a pinch valve, blood was periodically supplied into a microfluidic channel for 20 s (i.e., T_on_ = 20 s, and T_close_ = 280 s) during each period. In other words, additional experiments were conducted using the same protocol as the proposed method, except that a disposable suction pump was replaced with syringe pump. Figure 2C(a) shows temporal variations of averaged image intensity (<I>) (i.e., syllectogram), depending on a syringe pump and a proposed pump. Here, the cavity volume (V_cavity_) of a proposed suction pump was set to V_cavity_ = 0.2 mL. To evaluate performance variations between a syringe pump and the proposed pump, two indices (AI and EAI) were calculated by integrating the syllectogram for a specific time (i.e., t_s_ = 250 s). As shown in Figure 2C(b), variations of AI and EAI were obtained by varying the flow rates of the syringe pump. As a result, AI as a conventional aggregation index remained constant without respect to the flow rate of the syringe pump. But, EAI as a newly proposed index increased linearly depending on flow rates of syringe pump. Furthermore, when the syringe pump was set to Q = 0.5 mL/h, there was no significant difference between the disposable suction pump (i.e., V_cavity_ = 0.2 mL) and the commercial syringe pump (i.e., Q = 0.5 mL/h, withdrawal mode). 

From this result, the disposable suction pump is able to sufficiently deliver blood from the pipette tip to the microfluidic channel. In addition, the EAI is more effective to quantify variations of RBC aggregation owing to continuous ESR in the pipette tip.

### 3.2. Quantitative Evaluation of the Channel Width and Conical Pipette Tip Effects

To evaluate the saturation of image intensity depending on the numbers of RBCs existed in a specific microfluidic channel, the image intensity of blood flow was quantified by varying channel width (W) (W = 500 µm, 2000 µm, and 3000 µm), channel depth (h) (h = 50 µm and 100 µm), and hematocrit (H_ct_) (H_ct_ = 30%, 40%, 50%, 60%, 70%, and 80%). According to digital image processing for grayscale optical images, the image intensity is 1 for pure white (i.e., <I>_upper_ = 1) and 0 for pure black (i.e., <I>_lower_ = 0), respectively. Since the image intensity of a grayscale image has <I>_upper_ = 1 and <I>_lower_ = 0, the maximum dynamic range is calculated as ΔI = 1.

**Figure 3:**
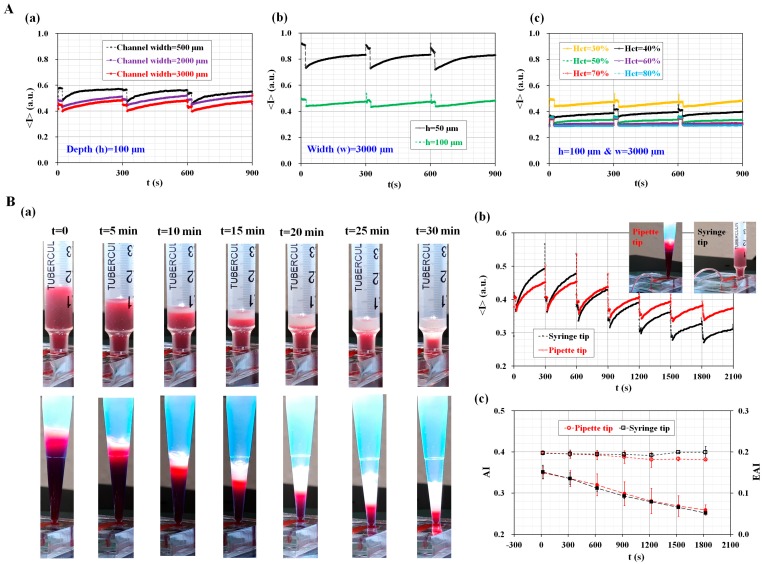
Quantitative evaluation of the effect of channel width (W), channel depth (h), hematocrit (Hct), and conical pipette tip on RBC aggregation measurement. (**A**) Characterization of the effect of channel width, channel depth, and hematocrit on performance; (**a**) Temporal variations of <I> with respect to channel width (W) (W = 500 µm, 2000 µm, and 3000 µm); (**b**) Temporal variations of <I> with respect to channel depth (h) (h = 50 µm and 100 µm); (**c**) Temporal variations of <I> with respect to hematocrit (Hct) (Hct = 30%, 40%, 50%, 60%, 70%, and 80%); (**B**) Quantitative comparison between syringe tip and conical pipette tip. (**a**) Sequential snapshot images over time (t) (t = 0, 5 min, 10 min, 15 min, 20 min, 25 min, and 30 min); (**b**) Temporal variations of <I> with respect to pipette tip and syringe tip; (**c**) Temporal variations of AI and EAI with respect to pipette tip and syringe tip.

As shown in Figure 3A(a), image intensity (<I>) was increased by decreasing channel width (or cross-sectional area). In other words, a microfluidic channel with a larger cross-sectional area (S_L_) (i.e., S_L_ = 100 × 3000 µm^2^) has a lower image intensity compared with a microfluidic channel with a shorter cross-sectional area (S_s_) (i.e., S_s_ = 100 × 500 µm^2^). Here, the hematocrit of the blood was fixed to 30% by adding normal RBCs into autologous plasma. From this result, image intensity varied depending the microfluidic channel width, especially at consistent hematocrit levels (i.e., H_ct_ = 30%). For a microfluidic channel with a wider channel width (i.e., channel width = 3000 µm), the maximum value of image intensity was much lower than the upper threshold value. Next, temporal variations of image intensity were obtained by varying channel depth. Here, channel width was fixed at 3000 µm. The hematocrit of the blood was fixed to 30% by adding normal RBCs into autologous plasma. As shown in Figure 3A(b), a low-depth channel (i.e., h = 50 µm) has a higher value of image intensity, compared with a high-depth channel (i.e., h = 100 µm). In other words, the image intensity of a low-depth channel was significantly increased to saturation value (i.e., <I>_upper_ = 1). Thus, the light source level of an optical microscope should be adjusted in order to avoid saturation in image intensity. At last, variations of image intensity were measured by varying hematocrit levels (Hct) (H_ct_ = 30%, 40%, 50%, 60%, 70%, and 80%). Here, channel width (w) and channel depth (h) were fixed at w = 3000 µm and h = 100 µm, respectively. As shown in Figure 3A(c), when hematocrit levels were below 60% (i.e., H_ct_ < 60%), RBC aggregation contributed to increasing image intensity over time. But, when hematocrit levels were above 60% (i.e., H_ct_ > 60%), variations of image intensity were extremely small with an elapse of time. Thus, it is impossible to quantify RBC aggregation due to saturation in image intensity above 60% of hematocrit. From these results, it is found that the proposed method is able to detect variations of image intensity of blood flows sufficiently, especially under consistent conditions (w = 3000 µm, h = 100 µm, and Hct < 60%). 

To find out the effect of pipette tip on the saturation of image intensity, quantitative comparison was conducted with both a pipette tip and a syringe tip. A syringe tip was prepared by cutting a disposable syringe (1 mL) appropriately. In other words, the syringe tip had a constant cross-sectional area; however, a conical pipette tip had a variable cross-sectional area. As shown in Figure 3B(a), sequential snapshot images were captured at intervals of 5 min with respect to syringe tip and pipette tip. By opening or closing the pinch valve periodically, the blood volume in the pipette tip or syringe tip gradually decreased over time. In addition, the RBC-depleted layer (i.e., plasma) was expanded gradually above the RBC layer with an elapse of time. As shown in Figure 3B(b), temporal variations of image intensity (<I>) were obtained with respect to pipette tip and syringe tip. A pipette tip showed a larger decrease in minimum image intensity (<I>_min_), compared with a syringe tip. From this result, the conical shape of the pipette had an influence on RBC concentration at the tip. Thus, the pipette tip had a lower value of <I>_min_, compared with the syringe tip. For both tips, since their image intensity was much lower than the upper limit, the suggested method has a sufficient dynamic range for the effective measurement of RBC aggregations. Temporal variations of the two indices (AI, and EAI) were obtained by using the syllectogram. As shown in Figure 3B(c), both tips show very similar trends over time with respect to AI and EAI. Therefore, it leads to the conclusion that the conical pipette tip suggested in this study can be effectively used to monitor variations of RBC aggregation under hematocrit variations.

### 3.3. Quantitative Evaluation of the Hematocrit Effect

**Figure 4:**
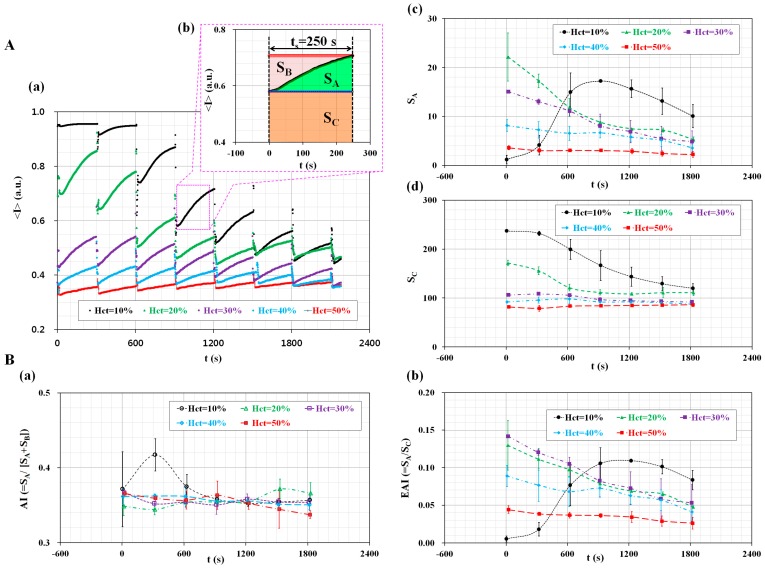
Quantitative evaluation of the effect of hematocrit (H_ct_) on RBC aggregation and ESR. (**A**) Temporal variations of S_A_ and S_C_ with respect to H_ct_; (**a**) Temporal variations of <I> with respect to H_ct_; (**b**) Three factors (S_A_, S_B_, and S_C_) are calculated by integrating the temporal variations of <I> for a specific duration of t_s_ = 250 s; (**c**) Temporal variations of S_A_ with respect to H_ct_; (**d**) Temporal variations of S_C_ with respect to H_ct_; (**B**) Temporal variations of AI and EAI with respect to H_ct_; (**a**) Temporal variations of AI with respect to H_ct_; (**b**) Temporal variations of EAI with respect to H_ct_.

The proposed method was employed to measure the effect of the hematocrit (H_ct_) on the measurement of RBC aggregation and ESR. The cavity volume of the disposable suction pump was fixed at V_cavity_ = 0.2 mL. The hematocrit of blood (H_ct_) was adjusted to H_ct_ = 10%, 20%, 30%, 40%, and 50% by adding normal RBCs into autologous plasma. By opening or closing the pinch valve, the blood flow was run for 20 s (T_open_ = 20 s), and stopped for 280 s (T_close_ = 280 s) for a single period of 300 s. As shown in Figure 4A(a), the temporal variations of <I> were obtained by varying the hematocrit from H_ct_ = 10% to H_ct_ = 50%. In addition, at intervals of 300 s, sequential microscopic images were obtained with respect to hematocrit levels, as shown in Figure S4. When hematocrit levels were below 30% hematocrit, the <I>_min_ for each period significantly decreased over time. However, when hematocrit levels were above 40% hematocrit, the <I>_min_ remained constant over time. As shown in Figure 4A(b), three factors (S_A_, S_B_, and S_C_) were calculated by integrating the temporal variations of <I> for a specific duration of t_s_ = 250 s. Using Equation (1), variations of S_A_ were obtained over time by varying the hematocrit levels. As shown in Figure 4A(c), except for the 10% hematocrit level, the variation of S_A_ decreased gradually over time. This result indicates that the hematocrit contributes to decreasing S_A_. Using Equation (3), variations of S_C_ were obtained over time by varying the hematocrit. As shown in Figure 4A(d), when hematocrit levels were below 20%, the variation of S_C_ significantly decreased over time. However, above 30% hematocrit, S_C_ remained constant over time. Like S_A_, S_C_ decreased when the hematocrit levels increased. As shown in Figure 4B, temporal variations of AI and EAI were obtained by varying the hematocrit levels. Figure 4B(a) shows the temporal variations of AI with respect to the hematocrit levels (H_ct_) (H_ct_ = 10%, 20%, 30%, 40%, and 50%). Except for the 10% hematocrit level, the temporal variations of AI remained constant, unrelated to the hematocrit. In other words, the conventional index (AI) cannot reveal distinctive differences with respect to the hematocrit. To compare with the AI, temporal variations of EAI were obtained by increasing the hematocrit levels. As shown in Figure 4B(b), the variation of EAI is very similar to S_A_. Since a continuous ESR occurs in a pipette tip under blood delivery, the variations of the hematocrit lead to a decrease in RBC aggregation. The variation of the hematocrit causes a significant decrease in EAI. According to previous studies, Kitamura et al. suggested that hematocrit levels contributed to varying RBCs aggregation through using ultrasonic backscattering signal [50]. In other words, scatter size and acoustic concentration were significantly increased with increasing hematocrit levels. However, ultrasonic backscattering signal was decreased at higher values of hematocrit [51,52]. Here, the backscattering signal had a negative magnitude in dB units. Yeom et al. measured RBCs aggregation using an ultrasonic signal (i.e., Echogenity) and image intensity [47], and found that echogenity increased with increasing hematocrit levels. Here, echogenity (i.e., E) had a positive magnitude in dB units. Two contrary results contributed to differences in the magnitude representation of the ultrasonic signal. In other words, backscattering had a negative sign, while echogenity had a positive sign. From this sign convention, it might be estimated that the difference in magnitude representation of the ultrasonic signals caused a change of trends in hematocrit levels. However, image intensity (i.e., L_1_) decreased with increasing hematocrit levels. In this result, trends of image intensity depending on hematocrit levels were very similar to the present result. In other words, the microscopic images of blood flows varied depending on light transmission through RBCs in microfluidic channels. Image intensity becomes dark, and is decreased at higher hematocrit levels. According to our previous work [28], an image intensity-based measurement technique suggested that RBC aggregation decreased as hematocrit levels increased. The present study gives very similar trends in variations of image intensity with respect to hematocrit levels. From previous studies, except for the ultrasonic-related result of Yeom et al. [47], ultrasonic backscattering signal and image intensity simultaneously decreased at higher hematocrit values. Thus, our suggested method shows similar trends to the ultrasonic system, depending on hematocrit levels. From this demonstration, the proposed method is able to effectively measure the variations in RBC aggregation owing to continuous ESR in a pipette tip. Since AI remains constant without the hematocrit, AI cannot be applied to monitor variations in the ESR. Instead of the AI, the EAI can be effectively employed to monitor the variation of RBC aggregation and the ESR.

### 3.4. Simultaneous Measurement of RBC Aggregation and ESR

Finally, the proposed method was applied to detect variations in RBC aggregation owing to a continuous ESR in the pipette tip. The disposable suction pump was prepared by setting the cavity volume with V_cavity_ = 0.2 mL. The hematocrit level of the blood was adjusted to H_ct_ = 30%. To stimulate RBC aggregation and ESR, two different samples of blood were prepared by using a specific dextran solution (i.e., aggregated blood) and heat treatment condition (i.e., thermally-induced hardening). 

**Figure 5:**
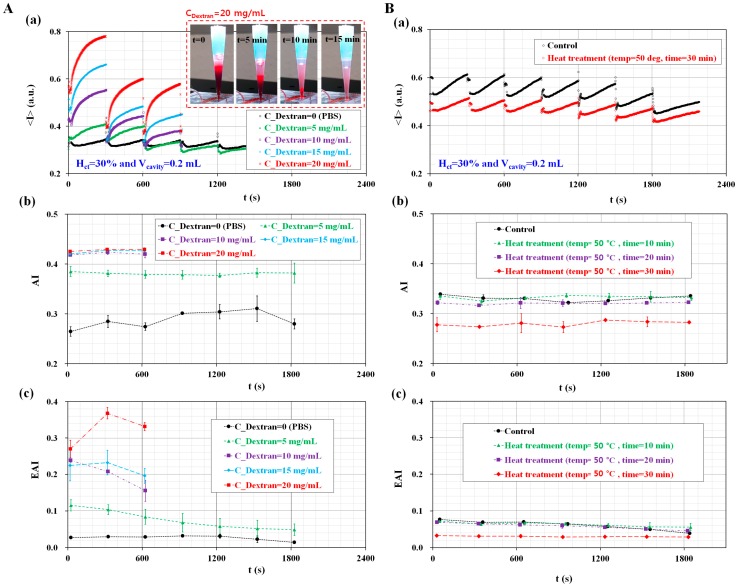
Quantitative evaluation of the proposed method for detecting RBC aggregated blood and thermally-induced blood. (**A**) Variations in ESR and AI by varying concentrations of dextran concentration; (**a**) Temporal variations of <I> with respect to dextran concentration (C_Dextran_) (C_Dextran_ = 0 mg/mL, 5 mL/h, 10 mg/mL, 15 mg/mL, and 20 mg/mL). For blood with 20 mg/mL dextran solution, the inset shows variations in the RBC-depleted regions (or plasma regions) in the pipette tip for the different time periods (t) (t = 0, 5 min, 10 min, and 15 min); (**b**) Temporal variations in the AI with respect to C_Dextran_; (**c**) Temporal variations in the EAI with respect to C_Dextran_; (**B**) Variations in RBC aggregation owing to ESR by varying heat treatment condition; (**a**) Temporal variations of <I> for the control blood and thermally-induced blood (30 min at a temperature of 50 °C); (**b**) Temporal variations of AI with respect to exposure time (t) at a temperature of 50 °C (t = 0, 10 min, 20 min, and 30 min); (**c**) Temporal variations in the EAI with respect to exposure time (t) at a temperature of 50 °C.

First, the aggregated blood was prepared by adding normal RBCs into a specific concentration of dextran solution (C_Dextran_) (C_Dextran_ = 0, 5 mg/mL, 10 mg/mL, 15 mg/mL, and 20 mg/mL). Figure 5A shows the effect of the dextran solution on variations in the AI and EAI over time. As shown in Figure 5A(a), the variations in <I> were obtained by increasing the concentration of the dextran solution. For aggregated blood with C_Dextran_ = 10, 15, and 20 mg/mL, the blood flow was continued up to 900 s. The variations of <I> were quantified for 900 s. However, for the aggregated blood with C_Dextran_ = 0 and 5 mg/mL, the variations of <I> were quantified for approximately 1800 s, because the blood flow continued for approximately 1800 s.

For the aggregated blood with C_Dextran_ = 20 mg/mL, the inset of Figure 5A(a) shows the variations in the RBC-depleted regions (or plasma region) in the pipette tip for a time period (t) (t = 0, 5 min, 10 min, and 15 min). Owing to the continuous ESR, the plasma region expanded gradually over time. After 900 s, only plasma existed in the pipette tip. As shown in Figure 5A(b), the temporal variations of the AI were obtained with respect to C_Dextran_. As a result, the AI is increased by elevating the concentration of dextran solution (C_Dextran_) from C_Dextran_ = 0 to C_Dextran_ = 10 mg/mL. However, for the aggregated blood above C_Dextran_ = 10 mg/mL, the AI remained constant over time. This result indicates that the AI is not effective for detecting aggregated blood with a dextran solution over 10 mg/mL. Figure 5A(c) shows the temporal variations of the EAI with respect to C_Dextran_. Unlike AI, the EAI increased gradually by elevating the concentration of the dextran solution. In addition, the EAI decreased gradually over time. However, the control blood (C_Dextran_ = 0) has a constant value of EAI = 0.03 over time, and thus, the control blood does not contribute to the variation of ESR in the pipette tip.

Second, the proposed method was employed to investigate the effect of high temperature on the RBC aggregation and ESR. The blood was prepared by adding normal RBCs in autologous plasma, and was exposed to the temperature of 50 °C for a specific time (t) (t = 0, 10 min, 20 min, and 30 min) in a convection oven [53,54]. Here, because the structure of plasma is reversibly changed below a temperature of 65 °C [55], the mechanical properties of the RBC membrane were varied by the heat treatment condition. After that, the blood was cooled down to the temperature of 25 °C. Figure 5B shows the variations in the AI and EAI with respect to the heat treatment condition. For the control and thermally-induced blood (50 °C × 30 min), the variations of <I> were obtained over time, as shown in Figure 5B(a). Compared with the control blood, the thermally-induced blood leads to a decrease of <I>_min_ and VA of <I> for each period. As shown in Figure 5B(b), temporal variations of the AI were obtained with respect to exposure time (t) (t = 0, 10 min, 20 min, and 30 min) at the temperature of 50 °C. Here, t = 0 denotes the control blood. Compared with the control blood (t = 0), the thermally-induced blood (t = 30 min) caused a significant decrease of AI. In addition, the AI remained constant over time. As shown in Figure 5B(c), temporal variations of the EAI were obtained with respect to exposure time (t) at the temperature of 50 °C. Below t = 20 min, the exposure time (t) does not contribute to variations of the EAI. The EAI gradually decreased over time. Since the continuous ESR in a pipette tip leads to an increase in the hematocrit levels, the RBC aggregation decreased over time. However, at t = 30 min, the EAI significantly decreased compared with the control blood (t = 0). In addition, the EAI remained constant over time. According to the previous work [1], the elasticity of blood significantly decreased from t = 20 min to t = 30 min at the temperature of 50 °C. In other words, when blood was exposed to the heat-treatment condition (50 °C × 30 min), the mechanical properties of the RBC membrane were degraded. Thus, thermally-induced blood caused a decrease in the two indices (AI and EAI). 

From this demonstration, the proposed method is able to effectively measure variations of a base solution (i.e., dextran solution) and the RBCs (i.e., thermally-induced hardening). In addition, the EAI can be effectively applied to evaluate variations in RBC aggregation and ESR. 

## 4. Conclusions

In this study, a microfluidic-based measurement technique was suggested to evaluate temporal variations of RBC aggregation under hematocrit variations owing to a continuous ESR. To demonstrate the proposed method, a microfluidic device was designed with an inlet, outlet, and single microfluidic channel. A pipette tip was tightly fitted into the inlet port. The disposable suction pump was composed of a disposable syringe (~1 mL), a specific spacer, and a pinch valve. The suction pump was connected to the outlet port with a polyethylene tube. After dropping blood (~0.2 mL) into the pipette tip, the blood was supplied into the microfluidic channel by releasing the pinch valve. 

To demonstrate the proposed method, the dynamic characterization of the disposable suction pump was first quantitatively measured by varying two factors, including hematocrit levels, and the cavity volume of the proposed pump. Since blood velocity (<U>) was increased by enlarging the cavity volume, and was gradually decreased over time, the disposable suction pump was able to sufficiently deliver blood from the pipette tip to the microfluidic channel. Next, variations in RBC aggregation and ESR were quantified by varying the hematocrit levels. The conventional aggregation index (i.e., AI) remained constant, unrelated to the hematocrit values. However, the EAI, as a newly suggested index, significantly decreased with respect to the hematocrit values. Thus, the EAI was more effective for monitoring RBC aggregation under hematocrit variations owing to continuous ESR. Finally, the proposed method was employed to detect variations in RBC aggregation and ESR by varying the base solution or RBC membrane stiffness. In other words, bloods were prepared by using a specific dextran solution and fixed RBCs with a heat-treated condition. The EAI gradually increased when the concentration of the dextran solution increased. In addition, thermally-induced blood caused to decrease the EAI.

From this experimental demonstration, the proposed method is able to effectively measure variations in RBC aggregation owing to continuous ESR in a pipette tip. In addition, the EAI can be effectively applied to evaluate the variation of RBC aggregation and ESR simultaneously. As a limitation of this study, the consecutive images were captured with a microscope equipped with a high-speed camera. Because of the bulk-sized and high-cost facility required, this method has limitations for its practical applicability. In the near future, to improve this issue, the image acquisition platform should be newly devised by using either a portable smartphone camera or a photo-sensitive sensor. In addition, this proposed method will be applied to evaluate variations in the biophysical properties of blood extracted from an animal model with cardiovascular diseases.

## Supplementary Materials

The following are available online at http://www.mdpi.com/1424-8220/17/9/2037/s1, Figure S1, Preparation of three spacers to secure cavity volume (V_cavity_). In addition, Figure S2, Variations of AI and EAI with respect to hematocrit (H_ct_) and specific duration of time (t_s_). Figure S3, Sequential snapshot images captured with smartphone-based camera at intervals of 30 s after clamping the tube with pinch valve. Figure S4, Sequential microscopic images obtained at intervals of 300 s with respect to hematocrit.
