# Supplementary material for: Microfluidic-Based Measurement Method of Red Blood Cell Aggregation under Hematocrit Variations
Source: Sensors (Basel). 2017 Sep 6;17(9):2037. doi: 10.3390/s17092037 (PMC5620946; doi:10.3390/s17092037)
Supplement: Supplementary file 1 [file sensors-17-02037-s001.pdf]

# Supplementary Materials

# Microfluidic-Based Measurement Method of Red Blood Cell Aggregation under Hematocrit Variations


**Yang Jun Kang**

Department of Mechanical Engineering, Chosun University, 309 Pilmun-daero, Dong-gu, Gwangju 61452, Korea; yjkang2011@chosun.ac.kr; Tel.: +82-62-230-7052; Fax: +82-62-230-7055


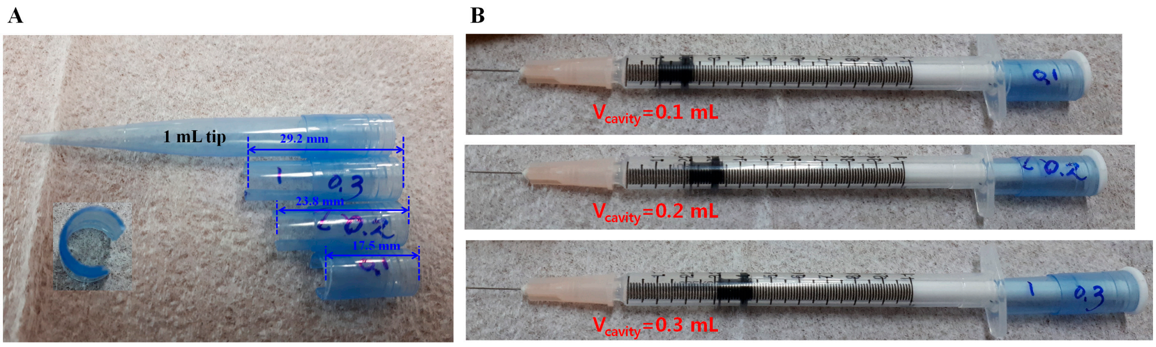


**Figure S1. (A)** Preparation of three spacers to secure cavity volume ($V_{cavity}$). The spacer for the corresponding cavity volume was cut at **(a)** L=17.5 mm ($V_{cavity}$=0.1 mL), **(b)** L=23.8 mm ($V_{cavity}$=0.2 mL), and **(c)** L=29.2 mm ($V_{cavity}$=0.3 mL) using a 1 mL pipette tip. The round wall of a spacer was partially cut. **(B)** A disposable suction pump with respect to cavity volume ($V_{cavity}$) ($V_{cavity}$=0.1 mL, 0.2 mL, and 0.3 mL).

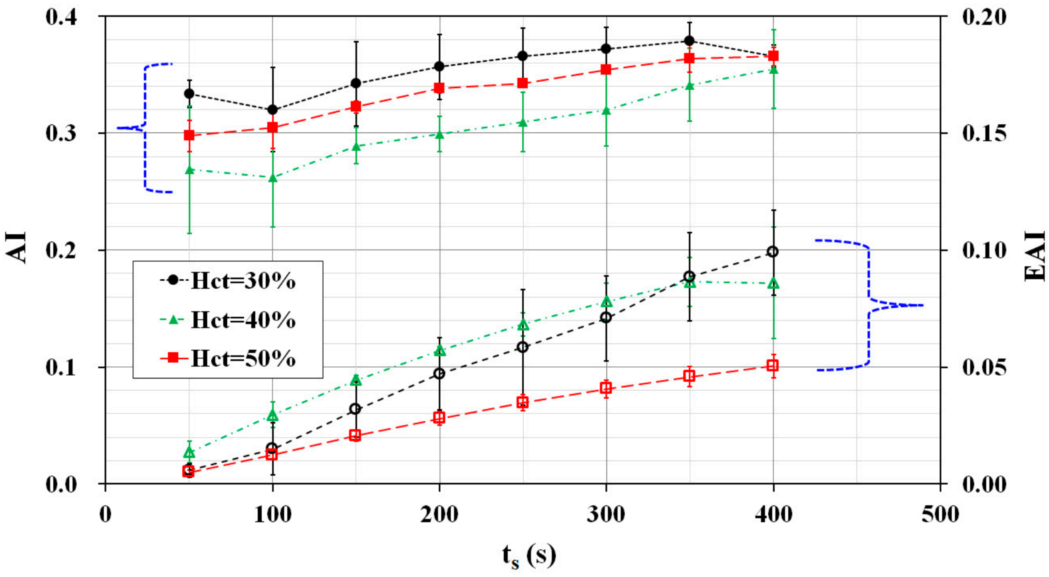


**Figure S2.** Variations of AI and EAI with respect to hematocrit ($H_{ct}$) and specific duration of time ($t_s$).

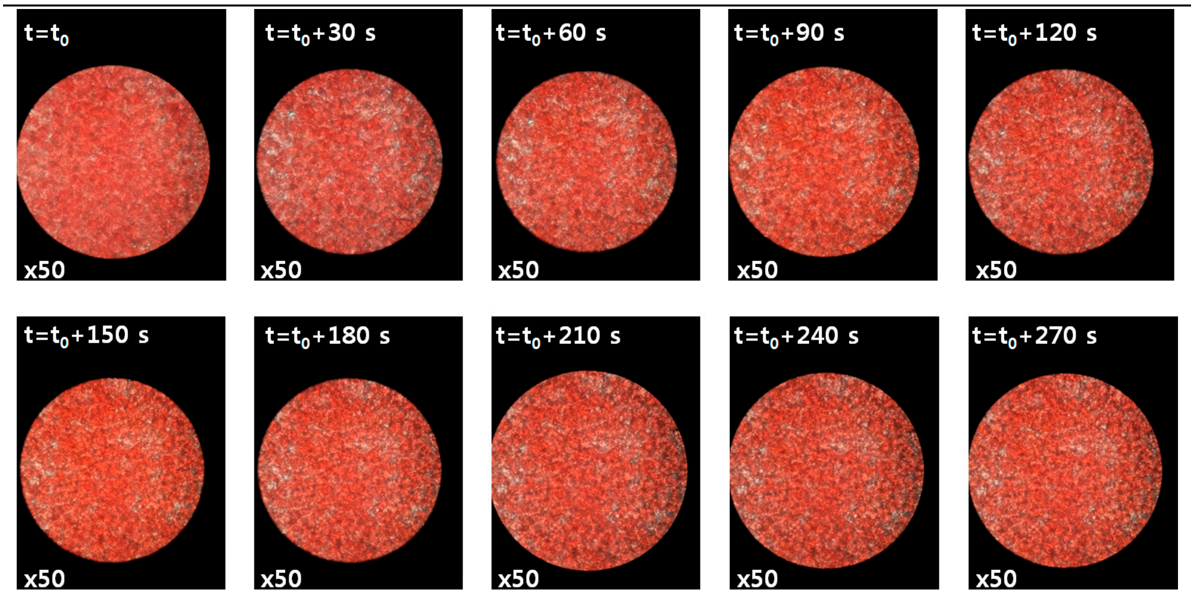


**Figure S3.** Sequential snapshot images captured with smartphone-based camera at intervals of 30 s after clamping the tube with pinch valve. Here, 50x objective lens (NA=0.5) was applied to monitor RBCs behaviors in details. RBCs-depleted regions (i.e., plasma) were expanded with an elapse of time. Since RBCs were distributed as multiple layers in depth direction, it is impossible to see rouleaux formation clearly.

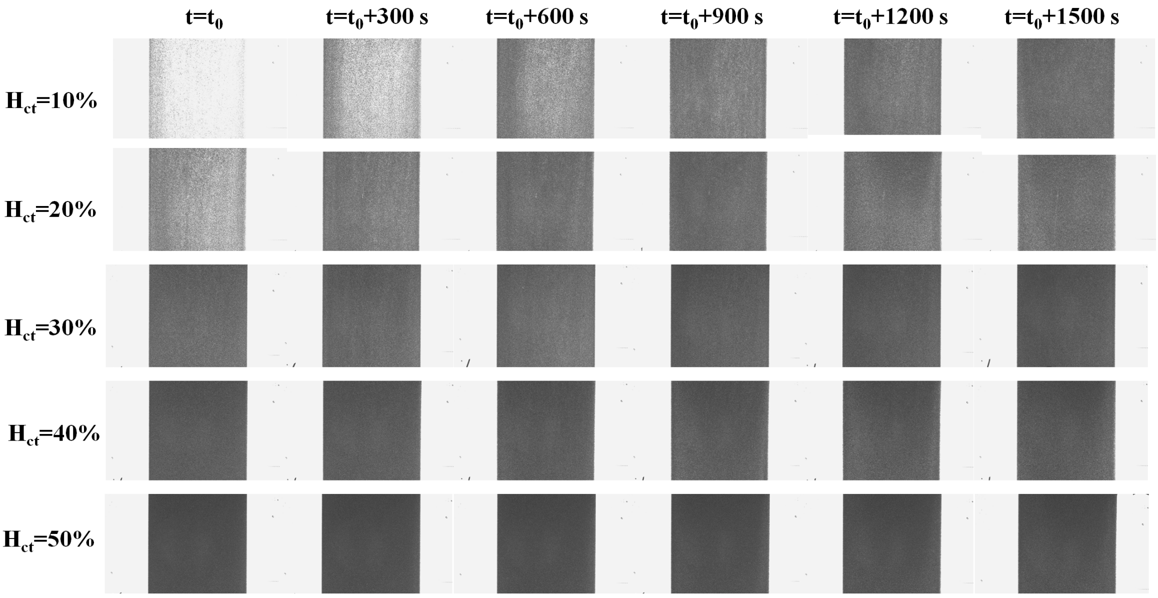


**Figure S4.** Sequential microscopic images obtained at intervals of 300 s with respect to hematocrit ($H_{ct}$) ($H_{ct}$=10%, 20%, 30%, 40%, and 50%). From these sequential images, image intensity was deceased over time. This results indicates that hematocrit variations in the conical pipette tip cause to decrease image intensity over time.
